# New Appliances and Things Medical

**Published:** 1906-02-03

**Authors:** 


### NEW APPLIANCES AND THINGS MEDICAL.

[We shall be glad to receive, at our Office, 28 & 29 Southampton Street, Strand, London, W.C., from the manufacturers, specimens of all new preparations and appliances which may be brought out from time to time.]

### WOLFE'S SCHIEDAM AROMATIC SCHNAPPS.

(UDOLPHO WOLFE, 22 BEAVER STREET, NEW YORK.)

THE sample before us may be regarded as a specially pure variety of gin. The therapeutic value of gin in certain disorders of the urinary system must be regarded as an established fact. Gin, as everyone knows, is usually made from a highly rectified spirit—that is, a relatively pure ethyl alcohol, to which is added oil of juniper and often other aromatic substances. The gin before us claims to be made from specially pure alcohol, in fact, from pure ethylic alcohol, there being in it no admixture of the so-called higher alcohols (fusel oil). Not only on account of its alcoholic moiety does this gin claim special purity, but also on account of its aromatic constituent. The oil used is the oil of the Italian juniper, and not that of the common juniper. Further, of the two oils which can be obtained from this plant the lighter alone is used. The product is exceedingly palatable, and in those cases in which gin is indicated it is to be recommended.

